# Physical activity of elderly patients with rheumatoid arthritis and healthy individuals: an actigraphy study

**DOI:** 10.1186/s13030-015-0046-0

**Published:** 2015-10-05

**Authors:** Toshihide Hashimoto, Kazuhiro Yoshiuchi, Shyuji Inada, Kenji Shirakura, Naoki Wada, Kimihiko Takeuchi, Masatoshi Matsushita

**Affiliations:** Department of Rehabilitation, Gunma University, 3-39-15 Syowa, Maebashi, Gunma 371-8511 Japan; Department of Stress Sciences and Psychosomatic Medicine, The University of Tokyo, 7-3-1 Hongo, Bukyo-ku, Tokyo 113-8655 Japan; Department of Orthopedics, Isesaki Fukushima Hospital, 556-2 Kashima, Isesaki, Gunma 372-0015 Japan

## Abstract

**Background:**

Most people with rheumatoid arthritis (RA) are physically inactive. An accelerometer worn on the waist has been used to evaluate physical activity in people with chronic conditions. It is useful for evaluating moderate to vigorous activity, although it tends to underestimate light or mild activities such as housework or family duties. An accelerometer worn on the wrist (i.e., actigraph) has recently been used to capture daily physical activity in inactive individuals. The purposes of this study were to investigate physical activity measured by an actigraph in patients with RA and in healthy individuals and to investigate the association between actigraphic data and self-reported physical function.

**Methods:**

The subjects were 20 RA patients and 20 healthy individuals. All participants wore an actigraph on their wrist for 6–7 consecutive days. They also completed the Health Assessment Questionnaire disability index (HAQ-DI) and the Medical Outcomes Study (MOS) 36-item short form health survey (SF-36). We extracted three parameters from the actigraphic data: mean activity count (MAC), peak activity count (PAC), and low activity ratio (LAR). These three parameters were compared between the RA patients and healthy individuals and with the self-reported questionnaires.

**Results:**

The MAC was significantly lower and the LAR was significantly higher in RA patients than in healthy individuals. The PAC was not different between the two groups. The LAR was negatively correlated with the MAC for the RA patients and for the healthy individuals. The decrease ratio of the LAR with the increase of the MAC for the RA patients was twice that of the healthy participants. In the RA patients, the LAR was significantly and moderately correlated with the HAQ-DI score and two dimensions of the SF-36 (i.e., “physical functioning” and “bodily pain”).

**Conclusion:**

Investigation of the proportion of low activity count using an actigraph may be useful to identify characteristics of the physical function in RA patients.

## Background

Rheumatoid arthritis (RA) is a chronic, systemic, progressive inflammatory disease characterized by swelling, pain, and deformity of the joints. RA affects synovial joints, many tissues, and organs. As a result, patients with RA experience functional disability because of multiple joint pain, general symptoms (e.g., anemia, fatigue, low-grade fever, or sleep disorders), and psychosocial disorders [1].

Recent studies indicate that most patients with RA are physically inactive. Based on data from 21 countries, only 13.8 % of patients with RA reported participating in regular exercise, which could provide health benefits in the general population [2]. The benefits of engaging in physical activity are well established for maintaining joint health and functional ability and for decreasing the severity of other chronic health conditions in RA patients [2, 3]. Even engaging in light-intensity and short-duration physical activities was protective against functional decline in older middle-aged adults with arthritis [4]. Therefore, it is important to evaluate accurately low-intensity activity and encourage as much daily activity as possible.

To evaluate the physical activity of individuals with chronic conditions, many previous studies examined subjective self-reported questionnaires. Questionnaires are inexpensive and easy to use in clinical studies; however, they have limitations, including recall bias such as overestimating aerobic activities and underestimating sedentary activities [5]. To complement subjective measures of physical activity, many studies have used an accelerometer worn on the waist. An accelerometer worn on the waist was useful in evaluating moderate to vigorous activity; however, it had difficulty in evaluating lower activity level of sedentary persons because of its inability to detect the arm movements [6, 7] of persons such as RA patients [8].

An accelerometer worn on the wrist has recently been used to capture low-intensity activity in disabled individuals, patients with tension-type headache [9], patients with dementia of the Alzheimer type [10], women with osteoarthritis [11], depressed elderly patients with psychomotor symptoms [12], and adolescents with chronic pain [13]. However, there have been few studies of RA patients that used a wrist-worn accelerometer.

Therefore, the aims of the present study were (1) to investigate the difference in physical activity patterns between RA patients and healthy individuals by use of a wrist-worn accelerometer and (2) to investigate the association between objective data by a wrist-worn accelerometer and subjective self-report data of physical function. Based on previous literature reports on RA patients using an accelerometer worn on the waist or the upper arm, we hypothesized that RA patients would demonstrate lower levels of physical activity compared to healthy individuals and that there would be a modest correlation between objective data by a wrist-worn accelerometer and subjective self-report data of physical function [14, 15].

## Methods

### Participants and procedures

Participants were RA patients under medical treatment who were scheduled to start a biologic agent in Gunma University Hospital (Gunma Prefecture, Japan) or Isesaki Fukushima Hospital (Gunma Prefecture, Japan). They were recruited when they visited their physicians as outpatients. The eligibility criteria of the RA patients were the following: (1) they fulfilled the American Rheumatism Association 1987 revised Criteria for RA [16]; (2) they were not experiencing an acute inflammatory episode; (3) they were 60 years or older; and (4) they were able to perform basic self-care activities in their home. Of the 23 patients met the eligibility criteria, two declined to participate because of scheduling conflicts and one was eliminated who could not wear the accelerometer in accordance with the instructions, leaving 20 patients as study participants.

The medical information of the RA patients (e.g., disease duration, radiographic findings, previous operation history, and disease activity) was collected from their medical charts. A radiographic evaluation of the wrist was classified by Larsen’s grading, which was divided into six categories based on erosion of the cartilage or bone, narrowing of the joint space, or destruction of the bony outlines [17, 18]. Disease activity was measured by Disease Activity Score 28-C-reactive protein (DAS28-CRP), which is calculated by the number of tender and swollen joints (of 28 joints), the C-reactive protein (CRP) level, and the patient’s self assessment of disease activity using a 100-mm visual analog scale. The DAS28-CRP is a valid measure of disease activity in RA patients [19].

Healthy individuals were recruited as controls by bulletin boards and flyers in the affiliated medical institute. The eligibility criteria of the healthy individuals were the following: (1) they did not have RA or other collagen diseases; (2) they were 60 years or older; (3) they were not dependent on analgesics because of chronic pain; and (4) they were able to perform basic self-care activities in their home. Twenty-five healthy individuals applied to participate and 23 of these individuals met the eligibility criteria. One individual declined to participate because of a scheduling conflict, one individual could not wear an accelerometer in accordance with the device’s instructions, and one individual lacked data because of device malfunction. Twenty healthy study participants remained. Medications of the participants remained unchanged during the study period.

The procedure and materials were approved by the institutional review board of Gunma University Hospital (Gunma Prefecture, Japan) and Isesaki Fukushima Hospital (Gunma Prefecture, Japan). All study participants provided written, informed consent. The study period was from June 2010 to December 2012.

### Objective measures of physical activity

The objective physical activity of the participants was evaluated by the Actigraph Mini-Motionlogger (Ambulatory Monitors Inc., Ardsley, NY, USA), an omnidirectional accelerometer worn on the wrist. It contains a piezoelectric element with a sensitivity of 0.01 G/min. Zero-crossing mode was used and acceleration counts were accumulated for every epoch of 1 min.

All participants were instructed to wear the actigraph on the nondominant wrist for 24 h per day for 6–7 consecutive days. By filling out a daily log time sheet, they were also instructed to identify their bedtime, wake-up time, and the time that the device was removed (e.g., during bath time).

### Subjective measures of physical function

On the final day of the actigraphic survey, subjective physical function was assessed by questionnaires; Health Assessment Questionnaire disability index (HAQ-DI), which is a widely used and validated tool to quantify functional ability in patients with RA. It was calculated as the mean of the highest score in 8 dimensions: dressing, rising, eating, walking, hygiene, reach, grip, and usual activity. The scores range from 0 (i.e., no disability) to 3 (i.e., severe disability) [20]. The Japanese version of the Health Assessment Questionnaire is also reported to be valid and reliable for measuring the functional status of Japanese RA patients [21].

### Health-related quality of life

Health-related quality of life was measured by the Medical Outcomes Study (MOS) 36-item short-form health survey (SF-36). The SF-36 is a general quality of life questionnaire that consists of eight dimensions: physical functioning, role-physical, bodily pain, general health perception, vitality, social functioning, role-emotional, and mental health. The score ranges from 0 to 100; a higher score indicates a better quality of life [22]. In clinical tests of validity, the subscales of the SF-36 Japan version were valid in discriminating between groups with and without a severe physical condition [23].

### Data analysis

We defined 12 h between 8:00 am and 8:00 pm as the investigation period. Participants differed in activities after just getting up and just before bedtime that were influenced by their life style or home environments (e.g., cooking, bathing, or drinking alcohol). Therefore, we excluded the hours before 8:00 am and after 8:00 pm from the investigation period. Data from the actigraphs were analytically filtered to identify nonwearing periods and days without sufficient wear time by use of the following method. When an actigraph was not worn, judged by the daily log, or when 10 or more contiguous minutes of 0 activity count were found, these periods were excluded from the further analysis [11, 13]. A day with 10 wearing hours or more was defined as a valid day [24]. Only participants who had four valid days or more of monitoring were included in the analysis [25]. The mean (standard deviation [SD]) of valid days for all participants was 6.0 (0.9).

We extracted three parameters from the actigraphic data based on the method by Wilson et al. [13], who assessed the physical activity of adolescents with and without chronic pain by actigraphy. The mean activity count (MAC) was calculated as the average value of all activity counts per minute during the investigation periods of all valid days. The peak activity count (PAC) was the highest number of all activity counts during the investigation periods. The low activity ratio (LAR) was calculated as the proportion of the numbers less than 40 of all activity counts per minute during the investigation periods [13].

### Statistical analysis

Parameters were compared between RA patients and healthy individuals. The correlations between each parameter and self-reported measures (i.e., the HAQ-DI and subscales of SF-36) were investigated. The statistical analysis was calculated using R version 2.13.1 (The R Foundation for Statistical Computing), based on the variables, the variances, and the distributions: Welch two-sample *t* test or the Wilcoxon rank sum test for comparing two groups; the Pearson’s product–moment test or the Spearman’s rank sum test for the correlating variables; the Fisher’s exact test for testing independence in the contingency tables; and analysis of covariance (ANCOVA) for modeling continuous and categorical variables. For all analyses, a p value less than 0.05 was considered significant.

## Results

### Sample characteristics

Table 1 shows the sample characteristics of the two groups in the present study. There were no differences in age, sex, body mass index, numbers of comorbidity, employment status, and family composition between the two groups. RA patients varied in disease duration, radiographic evaluation, and disease activity.

**Table 1:** Characteristics of participants

	RA patients (*n* = 20)	Healthy individuals (*n* = 20)	p
Age			
mean (SD)	69.4 (5.1)	69.6(6.6)	NS
Sex			
female - no. (%)	16 (80.0)	16 (80.0)	NS
Body mass index			
mean (SD)	21.4 (3.6)	23.4 (3.4)	NS
Comorbidity			
one or over - no. (%)	7 (35.0)	8 (40.0)	NS
Employment			
employed - no. (%)	4 (20.0)	7 (35.0)	NS
Family composition			
with children - no. (%)	9 (45.0)	7 (35.0)	NS
with spouse - no. (%)	9 (45.0)	9 (45.0)	
single - no. (%)	2 (10.0)	4 (20.0)	
Disease duration			
< 1y - no. (%)	5 (25.0)	-	
1y-5y - no. (%)	2 (10.0)		
≧ 5y - no. (%)	13 (65.0)		
Radiographic evaluation			
≦ Larsen III- no. (%)	9 (45.0)	-	
≦ Larsen IV- no. (%)	11 (55.0)		
Operation due to RA			
one or over - no. (%)	5 (25.0)	-	
DAS28-CRP			
mean (SD)	3.59 (1.22)	-	
mild (<2.7) - no. (%)	6 (30.0)		
moderate (2.7-4.1) - no. (%)	9 (45.0)		
high (>4.1) - no. (%)	5 (25.0)		

*NS* non-significance by Welch two sample *t* test or Fisher’s exact test

### Objective measures of physical activity

There were no significant differences for MAC, PAC, or LAR between male and female participants or between employed and non-employed participants. However, the participants living with children had lower MAC (Welch two-sample *t* test, t = 2.70, *p* < 0.05) and higher LAR (Welch two-sample *t* test, t = −2.33, *p* < 0.05) than the other participants. In RA patients, there were no significant differences in the disease activity for MAC, PAC, or LAR. However, the LAR tended to be higher in the RA patients with a long disease duration (≧5 years) and in the RA patients with advanced radiographic change (≧ Larsen IV) than in the other RA patients (Welch two-sample *t* test, t = −1.82, *p* = 0.085, effect size (EZ) = 0.58 and t = −1.67, *p* = 0.11, EZ = 0.53, respectively). The effect sizes were moderate.

**Fig. 1:**
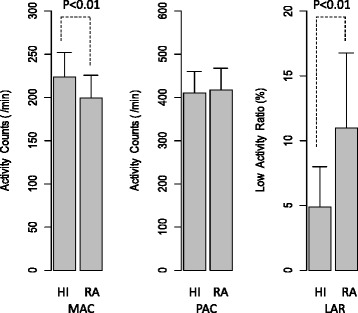
The mean (standard deviation) of the actigraphic measures. The bar plots indicate the mean (standard deviation [SD]) value of the mean activity count (MAC), peak activity count (PAC), and low activity ratio (LAR) for healthy individuals (HI) and for rheumatoid arthritis (RA) patients. In the RA patients, the MAC is significantly lower (Welch two-sample *t* test: t = 2.74, *p* < 0.01) and the LAR is significantly higher (Welch two-sample *t* test: t = −4.16, *p* < 0.01) than was found for the healthy individuals. By contrast, the PAC is not significantly different between the RA patients and the healthy individuals (Wilcoxon rank sum test: W = 183.5, *p* = 0.66). HI, healthy individuals; LAR, low activity ratio; MAC, mean activity count; PAC, peak activity count; RA, rheumatoid arthritis

Figure 1 shows the bar plots of the actigraphic parameters. The mean (SD) of the MAC was 199.2 (26.9) per minute in RA patients and 223.4 (28.8) per minute in healthy individuals. The mean (SD) of the LAR was 11.0 % (5.8 %) in RA patients and 4.9 % (3.1 %) in healthy individuals. In the RA patients, the MAC was significantly lower (Welch two-sample *t* test, t = 2.74, *p* < 0.01) and the LAR was significantly higher (Welch two-sample *t* test, t = −4.16, *p* < 0.01) when compared to the healthy individuals. The PAC was not significantly different between the RA patients and the healthy individuals (Wilcoxon rank sum test, W = 183.5, *p* = 0.66).

**Fig. 2:**
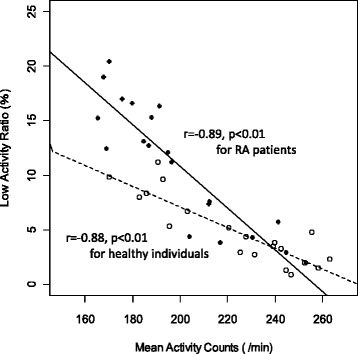
The relation between the MAC and LAR in RA patients and healthy individuals. The black dots represent RA patients and the open dots represent healthy individuals in the scatter plot of the MAC and the LAR. The LAR is inversely correlated with the MAC in both groups (Pearson’s product–moment correlation: r = −0.89, *p* < 0.01 for RA patients; r = −0.88, *p* < 0.01 for healthy individuals). In the analysis of covariance (ANCOVA) with MAC as the covariant, there were significant interaction terms between MAC and the groups (*p* < 0.01). The regression slope of the RA patients is twice that of the healthy patients (RA patients, −0.19; healthy individuals, −0.09). LAR, low activity ratio; MAC, mean activity count; RA, rheumatoid arthritis

Figure 2 shows the scatter plot of the MAC and LAR of the RA patients and healthy individuals. The LAR was negatively correlated with the MAC in both groups (Pearson’s product–moment correlation: r = −0.89, *p* < 0.01 for RA patients; r = −0.88, *p* < 0.01 for healthy individuals). In the analysis of covariance (ANCOVA) with MAC as a covariant, there were significant interaction terms between the MAC and the groups (*p* < 0.01). The regression slope of the RA patients was twice that of the healthy individuals (RA patients, −0.19; healthy individuals, −0.09). In the RA patients, the association between the MAC and LAR was significant, after controlling for age, disease duration, and disease activity (partial correlation coefficient = −0.19, adjusted R^2^ = 0.83, *p* < 0.01).

### Association between objective physical activity and subjective measures of physical function

Table 2 shows the median (inter-quartile range [IQR]) and mean (SD) of variables of the subjective measures (i.e., SF-36 and HAQ-DI) of RA patients and healthy individuals. The bivariate correlations (i.e., Spearman’s rank correlation *rho*) between the subjective measures and the actigraphic measures are calculated in Table 3.

**Table 2:** Median (IQR) and mean (SD) of subjective measures (SF-36, HAQ-DI) of RA patients and healthy individuals

	RA patients (*n* = 20)		Healthy individuals (*n* = 20)		
	Median (IQR)	Mean (SD)	Median (IQR)	Mean (SD)	p
SF-36					
Physical Functioning	50.0 (42.5)	50.0 (26.3)	90.0 (18.8)	83.0(18.9)	*
Role-Physical	59.4 (36.6)	56.6 (25.8)	96.9 (25.8)	86.1 (19.2)	*
Bodily pain	43.0 (23.3)	48.9 (20.6)	72.0 (28.5)	73.6 (21.1)	*
General Health Perception	45.0 (19.1)	44.2 (17.0)	71.0 (31.3)	73.1 (17.6)	*
Vitality	50.0 (20.4)	45.2 (15.2)	75.1 (25.0)	73.8 (17.6)	*
Social Functioning	68.8 (50.0)	66.9 (31.0)	100.0 (0.0)	94.1 (12.8)	*
Role-Emotional	58.3 (25.0)	61.9 (26.2)	100.0 (16.7)	90.4 (17.2)	*
Mental Health	60.0 (25.1)	62.3 (17.5)	90.0 (17.5)	85.3 (15.1)	*
HAQ-DI	1.00 (1.06)	1.31 (0.89)	0.00 (0.00)	0.09 (0.28)	*

*SF-36* MOS 36-item short-form health survey, *HAQ-DI* Health Assessment Questionnaire disability index, *IQR* Inter-quartile range, *SD* Standard deviation

*p < 0.01 by Wilcoxon rank sum test

**Table 3:** Spearman’s correlations between subjective measures (SF-36, HAQ-DI) and actigraphic measures

	RA patients (*n* = 20)			Healthy individuals (*n* = 20)		
	MCA	PAC	LAR	MAC	PAC	LAR
SF-36						
Physical Functioning	0.38	0.03	−0.45*	0.20	0.35	−0.06
Role-Physical	0.33	0.44	−0.31	0.39	0.55*	−0.26
Bodily Pain	0.35	−0.05	−0.47*	0.43	0.51*	−0.33
General Health Perception	0.26	−0.12	−0.21	−0.28	−0.26	0.22
Vitality	0.00	−0.15	−0.08	0.08	0.11	−0.14
Social Functioning	0.23	0.15	−0.27	0.02	0.00	−0.05
Role-Emotional	0.22	0.23	−0.23	0.04	0.06	−0.04
Mental Health	0.11	0.04	−0.13	−0.05	0.08	0.05
HAQ-DI	−0.52*	−0.08	0.58**	−0.05	−0.18	−0.14

*SF-36* MOS 36-item short-form health survey, *HAQ-DI* Health Assessment Questionnaire disability index, *MAC* Mean activity count, *PAC* Peak activity count, *LAR* Low activity ratio

* *p* < 0.05, **p < 0.01

In all dimensions, the SF-36 scores were significantly lower (Wilcoxon rank sum test, *p* < 0.01) in RA patients than in healthy individuals (Table 2). In the RA patients, the LAR was significantly correlated with “bodily pain” (Spearman’s rank sum test: *rho −*0.47, *p* = 0.03) and “physical functioning” (*rho* = −0.45, *p* = 0.04), that is the RA patients with better QOL in “bodily pain” and “physical functioning” had lower LAR. The MAC and PAC were not significantly correlated with any SF-36 subscale in the RA patients. In the healthy individuals, actigraphic parameters were not significantly correlated with the SF-36subscales, except PAC was correlated with “role-physical” (Spearman’s rank sum test: *rho* = 0.55, *p* = 0.01) and with “bodily pain” (Spearman’s rank sum test: *rho* = 0.51, *p* = 0.02) (Table 3).

The HAQ-DI score for the RA patients ranged 0–2.88, the median (IQR) score was 1.0 (1.06) while the 16 healthy individuals had a 0 score. The remaining four healthy individuals had scores of 0.13, 0.25, 0.50, and 1.25 (Table 2). In the RA patients, the HAQ-DI score was significantly correlated with the LAR (Spearman’s rank sum test: *rho* = 0.58, *p* = 0.007) and with the MAC (*rho* = −0.52, *p* = 0.018) (Table 3).

## Discussion

In this study, we observed two important clinical findings on the physical activities of elderly RA patients and healthy individuals. First, the MAC was significantly lower and the LAR was significantly higher in RA patients than in healthy individuals. The LAR was negatively correlated with the MAC for RA patients and for healthy individuals. The decrease ratio of the LAR with the increase of the MAC for RA patients was twice that of the healthy individuals. Second, in RA patients the LAR was significantly correlated with the HAQ-DI and with two dimensions (i.e., “physical functioning” and “bodily pain”) of the SF-36.

Compared to healthy individuals, the RA patients had a significantly lower MAC and higher LAR. However, the PAC was not significantly different between the two groups. In an actigraphy study of women older than 55 years with osteoarthritis (OA) and healthy individuals, Murphy et al. [11] reported that the average physical activity of the OA group was significantly lower than the average activity of the healthy group; however, the difference of the peak physical activity was not significant. On the other hand, in an actigraphy study [13] adolescent patients with chronic pain had both a lower mean activity level and a lower peak activity level than healthy controls. The authors of that study suggested that the mean activity level might be capturing the habitual level of physical activity and that the peak activity level might be an objective indicator of withdrawal from or nonparticipation in vigorous physical activities. We are aware of no published reports that have evaluated the physical activity of elderly RA patients using an actigraph. We supposed that RA patients would be similar to OA patients in an actigraphy investigation and that RA patients would have reduced habitual activities of daily life, compared to healthy individuals.

The LAR was negatively correlated with the MAC for RA patients and healthy individuals in the present study. The decrease ratio of the LAR with the increase of the MAC for RA patients was twice that of the healthy individuals. In an investigation of the correlation between “mean physical activity” (measured by the doubly labeled water method) and “low-intensity activity” using an accelerometer in healthy individuals, Meijer et al. [26] found that the physical activity level was inversely correlated with the percentage of time spent on low-intensity activity (i.e., lying, sitting, and standing) and that elderly individuals spent significantly more time on low-intensity activity than did younger individuals. Their finding of a correlation between the mean activity level and time spent in low-intensity activity was similar to our findings. In an investigation of the effect of regular exercise training on daily physical activity in healthy elderly people, Meijer et al. [27] found that nontraining physical activity on the training day was lower than the physical activity on the nontraining day: they suggested that the decrease in nontraining physical activity compensated for the training activity. In a qualitative study in RA patients, Repping-Wuts et al. [28] reported that RA patients had self-management strategies (e.g., pacing and rest, relaxation, and planning activity) as interventions for fatigue in their daily life. We supposed that elderly people would be trying to maintain their daily activity level by having periods of low-intensity activity and that elderly RA patients would have more low activity periods than the same aged healthy individuals.

The LAR had significant correlations with HAQ-DI and two dimensions (i.e., “physical functioning” and “bodily pain”) of the SF-36 in RA patients. In an investigation of the association between the Yale Physical Activity Survey (YPAS) and waist-worn accelerometer data in RA patients, the YPAS Activity Dimensions Summary Index was significantly correlated with the average daily minutes of high-intensity activities, but was not significant with low-intensity activities [15]. The authors of that study suggested that bias possibly might exist because low-intensity activities were not recalled as precisely as high-intensity activities in the self-reported questionnaire. They also mentioned that objective measurements were required to adequately capture low-intensity activities. In a study using an accelerometer worn on the upper arm in middle-aged women with RA, the correlations between the accelerometer data (e.g., energy expenditure and number of steps) and parameters related to physical function (e.g., HAQ, self-selected gait speed, timed chair rise, single leg stance test) ranged from low to moderate. After controlling for social and biomedical characteristics, only the HAQ score remained significantly associated with the number of steps measured by an accelerometer [14]. In the present study, the HAQ-DI score was significantly associated with the MAC and LAR in RA patients. Furthermore, the LAR was significantly associated with “physical functioning” and “bodily pain” of the SF-36, which contributes to the physical component score [29]. We believe that the investigation of low-intensity activities using a wrist-worn actigraph is a useful complement to the self-reported questionnaire for physical function.

The definition of the low-intensity activities is an important issue in the present study. The threshold of sedentary or low activities varied from 20 to 200 [13, 30–32], probably due to the differences of the measuring method, the definition of low activity, or the investigation population. We defined actigraphic counts less than 40 per minute as low-intensity activities based on the report of Wilson et al. [13], however, the mean activity counts and the peak activity counts of our participants were considerably lower than those of the participants in Wilson’s report. To identify the effect of this issue, we investigated the association between LAR and subjective physical function using counts less than 20 per minute for the RA patients. The LAR maintained significant correlations with “bodily pain” and “physical functioning” of SF-36 and HAQ-DI (Data are not shown). Furthermore, we investigated the nighttime activities of RA patients, which were activity counts from bedtime to getting up, and did not include the term of awakening. The 90 percentile values of the nighttime activities in the RA patients varied from 10 to 60 (mean (SD); 38.9 (16.2)) per minute. We supposed that counts less than 40 per minute might indicate sitting or lying still (e.g., sleeping, resting or watching TV) and be available for the threshold of low-intensity activities.

The present study has several limitations. The sample size was small. Further investigation in a large population is required to confirm the findings of this report and to identify the association between actigraphic data and biosocial factors, which we could not consider in the present study. Our RA patients were under medical treatment and were scheduled to start a biologic agent; therefore, their disease activity and general condition may be comparatively well controlled. Healthy individuals willingly participated in the study of daily activity, and may be more active than average elderly people. Therefore, the results of the present study may not be generally applied to RA patients or to healthy adults.

## Conclusion

Investigation of the proportion of low activity count using a wrist-worn actigraph may be useful to identify characteristics of the physical function of RA patients and may apply to evaluation of the physical function of other disabled people.

## Abbreviations

ANCOVA: Analysis of covariance
CRP: C-reactive protein
DAS28-CRP: Disease Activity Score 28-C-reactive protein
EZ: Effect size
HAQ-DI: Health Assessment Questionnaire disability index
IQR: Inter-quartile range
LAR: Low activity ratio
MAC: Mean activity count
OA: Osteoarthritis
PAC: Peak activity count
RA: Rheumatoid arthritis
SD: Standard deviation
SF-36: Medical Outcomes Study (MOS) 36-item short-form health survey
YPAS: Yale Physical Activity Survey.
