# Erratum to: Physical activity of elderly patients with rheumatoid arthritis and healthy individuals: an actigraphy study

**DOI:** 10.1186/s13030-015-0047-z

**Published:** 2015-10-26

**Authors:** Toshihide Hashimoto, Kazuhiro Yoshiuchi, Shyuji Inada, Kenji Shirakura, Naoki Wada, Kimihiko Takeuchi, Masatoshi Matsushita

**Affiliations:** Department of Rehabilitation, Gunma University, 3-39-15 Syowa, Maebashi, 371-8511 Gunma Japan; Department of Stress Sciences and Psychosomatic Medicine, The University of Tokyo, 7-3-1 Hongo, Bukyo-ku, 113-8655 Tokyo Japan; Department of Orthopedics, Isesaki Fukushima Hospital, 556-2 Kashima, Isesaki, 372–0015 Gunma Japan

## Erratum

After publication of this article [1], two errors were noticed in Table 1 and also in Table 3.

In Table 1 in the Radiographic evaluation section, one of the characteristics was displayed as: ≦ Larsen IV- no. (%), when it should have been: ≧ Larsen IV- no. (%).

In Table 3, one of the headings was displayed as MCA, when it should have been MAC, to refer to the Mean activity count.

The correct version of both Table 1 (Table 1 here) and Table 3 (Table 2 here) are displayed below. Both Tables have also since been updated in the original article [1].

**Table 1:** Characteristics of participants

	RA patients (*n =* 20)	Healthy individuals (*n =* 20)	p
Age mean (SD)	69.4 (5.1)	69.6 (6.6)	NS
Sex female - no. (%)	16 (80.0)	16 (80.0)	NS
Body mass index mean (SD)	21.4 (3.6)	23.4 (3.4)	NS
Comorbidity one or over - no. (%)	7 (35.0)	8 (40.0)	NS
Employment employed - no. (%)	4 (20.0)	7 (35.0)	NS
Family composition			
with children - no. (%)	9 (45.0)	7 (35.0)	NS
with spouse - no. (%)	9 (45.0)	9 (45.0)	
single - no. (%)	2 (10.0)	4 (20.0)	
Disease duration			
<1y - no. (%)	5 (25.0)	-	
1y-5y - no. (%)	2 (10.0)		
≧5y - no. (%)	13 (65.0)		
Radiographic evaluation			
≦ Larsen III- no. (%)	9 (45.0)	-	
≧ Larsen IV- no. (%)	11 (55.0)		
Operation due to RA			
one or over - no. (%)	5 (25.0)	-	
DAS28-CRP			
mean (SD)	3.59 (1.22)	-	
mild (<2.7) - no. (%)	6 (30.0)		
moderate (2.7-4.1) - no. (%)	9 (45.0)		
high (>4.1) - no. (%)	5 (25.0)		

*NS* non-significance by Welch two sample *t* test or Fisher’s exact test

**Table 2:** Spearman’s correlations between subjective measures (SF-36, HAQ-DI) and actigraphic measures

	RA patients (*n =* 20)			Healthy individuals (*n =* 20)		
	MAC	PAC	LAR	MAC	PAC	LAR
SF-36						
Physical Functioning	0.38	0.03	−0.45*	0.20	0.35	−0.06
Role-Physical	0.33	0.44	−0.31	0.39	0.55*	−0.26
Bodily Pain	0.35	−0.05	−0.47*	0.43	0.51*	−0.33
General Health	0.26	−0.12	−0.21	−0.28	−0.26	0.22
Perception	0.00	−0.15	−0.08	0.08	0.11	−0.14
Vitality	0.23	0.15	−0.27	0.02	0.00	−0.05
Social Functioning	0.22	0.23	−0.23	0.04	0.06	−0.04
Role-Emotional	0.11	0.04	−0.13	−0.05	0.08	0.05
Mental Health						
HAQ-DI	−0.52*	−0.08	0.58**	−0.05	−0.18	−0.14

*SF-36* MOS 36-item short-form health survey, *HAQ-DI* Health Assessment Questionnaire disability index

*MAC* Mean activity count, *PAC* Peak activity count, *LAR* Low activity ratio

*p < 0.05, **p < 0.01
